# How Artificial Intelligence and New Technologies Can Help the Management of the COVID-19 Pandemic

**DOI:** 10.3390/ijerph18147648

**Published:** 2021-07-19

**Authors:** Davide Barbieri, Enrico Giuliani, Anna Del Prete, Amanda Losi, Matteo Villani, Alberto Barbieri

**Affiliations:** 1 Department of Neuroscience and Rehabilitation, University of Ferrara, Via Savonarola 9, 44121 Ferrara, Italy; davide.barbieri@unife.it; 2 Department of Biomedical, Metabolic and Neuroscience Sciences, University of Modena and Reggio Emilia, Via Del Pozzo 71, 41125 Modena, Italy; en.giuliani@gmail.com; 3 School of Anesthesiology and Intensive Care, University of Modena and Reggio Emilia, Via Del Pozzo 71, 41125 Modena, Italy; annadelprete@hotmail.it (A.D.P.); alberto.barbieri@unimore.it (A.B.); 4 Department of Anesthesiology and Intensive Care, Azienda USL Piacenza, Via Antonio Anguissola 15, 29121 Piacenza, Italy; mvillani@live.it

**Keywords:** artificial intelligence, new technologies, pandemic management, COVID-19

## Abstract

The COVID-19 pandemic has worked as a catalyst, pushing governments, private companies, and healthcare facilities to design, develop, and adopt innovative solutions to control it, as is often the case when people are driven by necessity. After 18 months since the first case, it is time to think about the pros and cons of such technologies, including artificial intelligence—which is probably the most complex and misunderstood by non-specialists—in order to get the most out of them, and to suggest future improvements and proper adoption. The aim of this narrative review was to select the relevant papers that directly address the adoption of artificial intelligence and new technologies in the management of pandemics and communicable diseases such as SARS-CoV-2: environmental measures; acquisition and sharing of knowledge in the general population and among clinicians; development and management of drugs and vaccines; remote psychological support of patients; remote monitoring, diagnosis, and follow-up; and maximization and rationalization of human and material resources in the hospital environment.

## 1. Introduction

In December 2019, the new virus SARS-CoV-2, causing the severe acute respiratory syndrome, emerged in China, and rapidly spread around the world with an exponential growth curve.

On 11 March 2020, the World Health Organization declared the outbreak a pandemic, named COVID-19, and, as of 8 June 2021, 173,271,769 total confirmed cases and 3,733,980 deaths had been reported in the world [1,2].

Before the development of a specific vaccine, many countries took some public health measures to prevent or diminish the possibility of contagion [3]. Currently, environmental measures—such as isolation, quarantine, social distancing, and community containment—and vaccination are the most commonly adopted means that can significantly reduce the effects of the outbreak [4,5].

The consequences of the COVID-19 pandemic are becoming more evident day after day. As the number of patients is rising, intensive care units (ICUs) are collapsing, while the number of victims is increasing [6,7]. Recession is damaging the world economy, education has been affected, and psychological distress is surging in the population of all the most affected countries [8,9]. Furthermore, healthcare systems require more resources to deal with the pandemic, for example, isolation wards for communicable diseases, departments for intensive care, specialized health workers, ventilators, diagnostic imaging devices, medical treatments, and protection devices [10].

In this context, artificial intelligence (AI) and new digital technologies can support institutions, medical staff, and stakeholders by facilitating the timely sharing of medical information and the clinical management of the pandemic, tracking transmission in real time and—perhaps most importantly—remotely monitoring positive patients [11]. Machine learning (ML), a subfield of AI, is increasingly being applied to the medical domain. In ML, computers iteratively learn from data without explicit rule-based programming, identifying patterns to support rational human decision-making [12,13]. AI and ML were instrumental in COVID-19 drug discovery and vaccine development [14].

The aim of this paper was to evaluate how AI and new technologies can facilitate clinical practice during the COVID-19 pandemic, especially in critical care settings, pointing out some of the main advantages and obstacles.

After this Introduction, where the topic of the paper is presented and the aim of the study declared, the Materials and Methods section contains the description and explanation of the adopted methodology for the selection of the relevant papers; in Results, the outcomes and major findings of the selected papers are summarized; finally, in the Discussion and Conclusions section, the findings are discussed in depth, especially for their implications on the management of the current wave of the pandemic and for the recommendations that can be elicited from them for the prevention of any eventual subsequent wave.

## 2. Materials and Methods

We conducted a literature review on new technologies that have been adopted to contain and monitor the COVID-19 pandemic. Consequently, we selected papers that are relevant to these specific issues, to support the discussion and draw possible conclusions.

The following keywords were used for a comprehensive search: “COVID-19 Map, COVID-19 Mobile apps, Lockdown, SARS-CoV-2 transmission, Contact tracing, Machine Learning, COVID-19 End-to-end system, Shared Decision Making, Forecasting outbreak, Medical Data, Video consulting, Artificial Intelligence, remote monitoring, telemedicine”.

The searched databases were PubMed and Web of Science. The selected papers included the following types: systematic review, original research article, editorial, narrative review, Cochrane review, e-book chapter.

## 3. Results

There are several opportunities for the adoption of new technologies against the COVID-19 outbreak. The Center for Systems Science and Engineering at Johns Hopkins University created a digital platform for sharing data related to the worldwide spread of the COVID-19 pandemic [1]. Data are updated daily on a hosting website and displayed on an electronic dashboard, publicly available. It shows a world map of cumulative confirmed cases, active cases, and incidence and case-fatality rate.

Several mobile apps have been developed in an attempt to limit the growing number of COVID-19 cases and deaths [15]: they have been adopted for information sharing (decreasing misinformation and confusion), risk assessment, self-management of symptoms (tracking COVID-19 symptoms and mental health of citizens), decision-making, contact tracing, home monitoring, and isolation. Thanks to its accessibility, acceptability, and ability to support social distancing, the remote monitoring of the psychophysical health of citizens forced to stay at home because of the restrictive measures, can play a fundamental role, considering the implication of the pandemic on psychological and physical health [16,17].

Several smartphone apps have been developed and adopted in many countries to monitor any violation of the quarantine, track the movements of positive COVID-19 subjects, and eventually identify new possible contacts. By means of Bluetooth (a radio-frequency technology that allows devices to exchange information at close range) and GPS geo-localization systems (which determine the position of a receiver through the use of satellites), it is possible to quickly identify the contacts of confirmed positive individuals and isolate them [18,19]. Most apps share information using Bluetooth, and this is convenient, because it is a very widespread technology. Another possibility, in addition to Bluetooth and GPS, is self-reporting by users, as the Chinese government has done. Asian countries, such as China, South Korea, and Singapore, are pioneers in developing and using tracking measures for fighting the COVID-19 epidemic [20]. The government of Singapore has created an app to monitor the compliance of positive or suspected individuals to quarantine measures. In South Korea, apps like Corona Map^®^ (Seul, Korea, governmental app) and Corona 100m^®^ (Seul, Korea, governmental app) have been developed by the government and/or private companies. These apps use the geolocation system to inform the users of the proximity of positive or suspected individuals [21]. The Chinese government has implemented a new function in two apps that were already commonly used by the population: Alipay^®^ (Alibaba Group, Hangzhou, China) and WeChat^®^ (Tencent Holdings Ltd., Shenzhen, China).

The function generates a Health Code associated with different colors (green, yellow, red) based on the risk of being infected [22].

The Australian COVIDSafe app can predict whether an app user has had close contact with a person testing positive for the COVID-19 swab test. In such a case, the app user will be contacted by health officials.

The United Kingdom’s contact-tracing app requires users to describe their symptoms and allows the health authorities to alert the person’s contacts. A team of the University of Oxford analyzed key parameters of the pandemic’s spread and developed a mathematical model of infection, which was then implemented in a contact-tracing app that builds a database of social contacts and immediately notifies proximity with positive subjects [21]. In June 2020, the UK government abandoned a centralized contact-tracing app and adopted the new Google–Apple framework for future COVID-19 contact-tracing software.

In the United States of America, some states developed contact-tracing apps for residents; for example, the Care 19 app in South Dakota (ProudCrowd, LLC, North Dakota, governmental) and the Healthy Together app (twenty inc., Dallas, TX, USA) in Utah and Florida. Last May 2020, Google and Apple jointly decided to release common application programming interfaces (APIs) that allow governments to develop their contact-tracing apps with the possibility of data exchange [20].

The app Dados do Bem was developed in Brazil to track COVID-19 symptoms: applying machine learning techniques, it was possible to provide a SARS-CoV-2 infection risk map of the city of Rio de Janeiro, representing a potential tool for decision-makers to refine disease-control strategies [20,22].

Home monitoring is one of the most interesting and helpful applications of new technologies under self-quarantine and self-isolation circumstances: for example, Li et al. developed Wi-COVID, a framework using a non-wearable technology and a WiFi signal generated by standard devices at home to monitor the respiratory rates (RRs) of COVID-19 patients and communicate it to healthcare providers in real time [23].

In Italy, a private initiative, with a public endorsement and a government call, developed the Immuni^®^ app (Bending Spoons, Milan, Italy, Governmental), which can be used on a voluntary basis. It is available since 1 June 2020. Immuni^®^ requires users to activate the notification of exposure to SARS-CoV-2. When the health structures and the national health system find a positive case, they enter a code in the system, with the consent of the subject. At this point, the system will send a notification to the users who were in close contact with the positive case [24]. This allows the users to contact their general practitioner promptly, in order to receive information on the steps to be taken. Immuni^®^ was developed in compliance with Italian and European legislations on personal data protection (GDPR). The system does not acquire names or any other elements that could identify the positive individuals or those who had any contact with them but uses alphanumeric codes (pseudonymization). The Bluetooth connection gives the users the possibility to choose whether and when to share information about their position, simply by activating or deactivating this option on the mobile phone.

In many cases, social distancing is the only measure applicable: the consequent isolation and personal protective equipment can hinder the communication process, so it becomes important to develop specific guidelines to assist healthcare workers in delivering effective shared decision-making (SDM) in order to customize the treatments of patients [25]. SDM is a management model that allows a two-way exchange of information between patient and doctor, in to optimize and personalize the provided care. Although the current pandemic has made this process more difficult, because of limited resources and social distancing, it is needed more than ever in this moment of great uncertainty caused by the spread of unreliable news [26,27].

Currently, ML is applied to different fields in the biomedical sciences. Its aim is to help the personalization of medical practice, so that it can be tailored for the patients [28]. In sport medicine, ML can be used for both performance and risk prediction [29,30]. Further, ML can identify molecular markers for cancer treatments, evaluate postoperative surgical outcomes, and supply an automated interpretation of an ECG or an automated detection of a lung nodule in a chest X-ray [31]. In all these cases, an ML model approximates a trained physician’s diagnosis with high accuracy [11,32]. ML can assist intensive care specialists in early detection of patient’s shock and septic conditions, including COVID-19 infections [33]. Epidemiologists may use ML forecasting capabilities in order to assess the spread of pandemics [34,35,36].

ML has extensive potential applications. For example, it can help researchers to develop drugs against COVID-19. ML-based algorithms support researchers in finding specific inhibitors of a biomolecule, such as a COVID-19 epitope [14]. Furthermore, ML can identify different COVID-19 viral sequences from various parts of the world and subsequently build a library of peptides that can be used to provide cross-protection against different COVID-19 variants [37].

It is also possible to use ML to develop vaccines containing an extensive repertoire of epitopes, such as stimulating a large number of different T cells to provide global protection. Furthermore, ML can help to reverse vaccinology to identify antigens of proteins essential for viral pathogenicity (for example for adhesiveness and cell invasion), in order to induce high protection [38,39].

ML and AI can contribute also to the distribution of the vaccine and to monitorvaccinated people. For example, Asgary et al. developed an ML model (turned to an online application) that can help mass vaccination planners to quickly monitor different types of drive-through mass vaccination facilities [40], while Bubar et al. used a mathematical model to compare age and serostatus-stratified prioritization strategies [41]. Furthermore, Mathieu et al. have realized a COVID-19 vaccination dataset, a regularly updated global public dataset that tracks the scale and rate of the vaccine rollout across the world [42].

Modern electronic medical record (EMR) systems use ML algorithms to provide clinical work support, offering the right information at the right time to the right people and, thus, making efficient use of the available resources [43].

Wireless wearable sensors are now adopted to monitor vital parameters non-invasively and continuously [44,45]. In some cases, such as chronic disease reviews, psychological counseling, or psychotherapy and triage, video consultation is sufficient, and therefore in-person visits may be avoided [46]. This may also be true in orthopedics, where patient selection is a crucial factor. In some cases, such as suture or staple removal, cast change etc., hands-on clinical examination is required [47]. In other cases, such as standard post-surgery checks of range of motion or wound condition, patients may be eligible for teleconsultation. If the condition is uncertain, remote triage can be adopted to decide whether an in-person visit is necessary [48,49].

Vascular surgery represents another opportunity for telemedicine. After gaining oral consent, vascular surgeons evaluated the patient via videoconference, asking for any new symptoms and performing a virtual physical examination. Then, they discussed medical management or surgical intervention and determined the appropriate treatment plan with the patient. Any medical question raised by the patient was also answered by the vascular surgeon [50].

In ophthalmology, video medicine has proved to be a precious tool. Clinicians reported that video consultations were particularly useful to see follow-up and post-operative patients and to more accurately triage and consult new referrals, who often had clinical information provided from the referrer. The resolution of the video image was suitable to assess eyelid position and movement, periocular swelling and hue, chemosis, ocular motility (including eliciting gaze-evoked pain), gross diplopia, facial asymmetry and function, and to perform assessment of reasonably sized eyelid lesions. When greater-resolution imaging was required, patients were usually able to take a good-quality photograph using a smartphone camera and digitally transfer this to the evaluating clinician for review, during the video consultation [51].

## 4. Discussion and Conclusions

The COVID-19 pandemic may be an opportunity to raise awareness on the importance of technological research applied to the medical field. New technologies may be used to spread medical information and knowledge, while physicians can play a role similar to that of influencers during the pandemic (some doctors have become very popular as opinion leaders) [52]. It would be interesting to exploit these possibilities, in order to spread healthy habits, disseminate medical culture, improve the understanding of medical decisions, and share the decision-making process [25]. The availability of open and big data gives scientists the possibility to perform studies reducing data collection time to a minimum, to assess the impact of new strategies, and to test new instruments to control the outbreak, including new applications. Thanks to a vast amount of data in electronic format, new hypotheses can be tested quickly, while results will be more reliable and significant from a statistical point of view.

Despite the premises and the potentiality of the apps mentioned above, the goal of containing the spread of the infection through tracking apps was only partially achieved. The reason for this is to be identified in the following points.

Countries are designing apps independently, and there are no standard guidelines: these smartphone apps are, in fact, health measures but are being developed without any risk assessment being carried out [21].

Each country still actually operates under different protocols, data organization, and legal rules, dispersing the effort to control the pandemic [15]. Smartphone users usually turn off Bluetooth when they are not using it, near other phone users, or to save battery charge. COVID-19 apps require users to keep Bluetooth always on, mainly when they are in public places. Due to the reopening of international travels, there is an increased risk of future waves of COVID-19: the international community should start working together to enable data sharing and data transfer between different contact-tracing apps. The isolation of contact-tracing apps will not contain the global spread of infectious diseases: all existing apps from foreign countries should communicate and exchange data [20].

The topic of privacy is still a challenge for new health-related technologies. The anonymization of data is a necessary prerequisite [53]. However, in the context of apps, we are not talking about “anonymization” of data but about “pseudonymization”: the pseudonymization process allows data to be identified only if combined with other information, stored separately, so it is possible that a subject may still be identified. In order to preserve privacy, a graph of anonymized interpersonal interactions can be adopted, to show transmission vectors of contagion or contact points [54,55].

Since SARS-CoV-2 is a communicable disease, contact tracing is one of the core strategies to be adopted in order to minimize transmission. New digital technologies can help in monitoring the COVID-19 pandemic and in evaluating exposure risk by means of a geographic information system.

Forwarding recommendations to individuals at risk, pandemics may be contained without the need for mass quarantines (lockdowns) that are detrimental to the society as a whole. By anonymously tracing the movements of confirmed positive subjects, early identification of their contacts becomes feasible. The subsequent steps would be contact isolation and early detection of symptoms.

Respect for privacy is essential in order to apply monitoring systems, such as GPS and smartphone apps, at large. As of 15 October 2020, Immuni^®^ has been downloaded 8 million times, but there is still reluctance to share movements even if anonymously, notwithstanding the fact that such movements and other personal information are shared more or less consciously daily through social media or access to various websites [56,57]. An online platform, in which every individual is identified by a nickname, may be developed to collect citizens’ health data, such as COVID-19 test results, symptoms, personal movements, etc. On the other hand, every time users download a new app, they give consent to share personal data with the owner of the app. This concept may be applied to health data as well. People should be aware that sharing medical data can lead to a significant improvement in public health, diminishing the risk of contagion and providing important information during an emergency.

In a medical condition such as SARS-CoV-2, ML can forecast a patient’s situation and eventually prevent cardiorespiratory failure, thanks to its predictive capabilities. ML can build a model based on the evolution of the subject’s biomedical parameters and, therefore, improve its predictions above the baseline since adoption.

In the context of a pandemic, where it is necessary to quickly transfer sensitive information about patients enrolled in experimental protocols to monitor the successful outcome of therapies or where it is necessary to transfer rapidly worsening patients from one health facility to another, technology plays a fundamental role. In order for communication between clinicians, from the same hospital or even more so from different hospitals, to be rapid and effective, it is important that the shared information is as essential, objective, and universally validated as possible. An EMR that meets these criteria would not only facilitate the daily work of the healthcare professional who compiles it, but it would also guarantee a quick and clear understanding of the clinical case by any colleague. In a pandemic, time is certainly a fundamental factor. Voice assistance to fill EMR may be an additional element to improve daily work: this “hands-off” or “hands-free” management would save time, especially in emergency or shortage conditions, or when the healthcare facility is saturated with patients.

**Figure 1:**
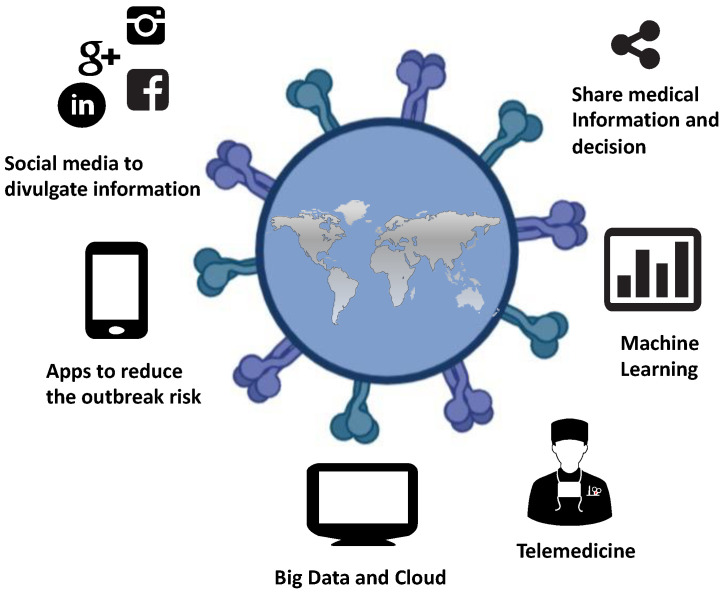
Artificial intelligence and new technologies against the COVID-19 pandemic.

Technological innovations have costs that are not always sustainable by all countries, especially where there is no basic infrastructure (internet connection, computers, personnel trained to use these resources). Health institutions should consider technology as an investment: even if it has starting costs, these are restored over time, in terms of increased productivity from a qualitative and quantitative point of view (Figure 1).

The process of technological innovation in the medical field should be achieved step by step: even small innovations are able to bring great benefits. The technological progress of the countries with the greatest economic resources can play a protective role toward countries with fewer resources, not only blocking the virus in the country in which it is detected but also giving other countries timely information to face the looming threat. Another advantage of technological investments in the COVID-19 emergency is the reduction of lockdown time and of new infections, which translates in terms of a faster economic recovery and reduced healthcare expenses [58].

The fact that healthcare facilities could be sources of contagion has drawn attention on new models of care that avoid face-to-face contact between clinicians and patients: there has been particular interest in telemedicine and video consultations. These instruments may be useful for clinicians who are self-isolating, for patients asking about COVID-19, for people with heightened anxiety, for those with symptoms related to SARS-CoV-2 (when a video consultation may reduce the need to visit a potentially contagious patient), and for older or immunosuppressed patients. However, video consultation of patients is unlikely to be appropriate for severely ill individuals, when a full physical examination or procedure cannot be deferred, or when comorbidities affect the patient’s ability to use technology.

The COVID-19 crisis revealed the limitations of our current structures to face a surge of acutely ill patients requiring hospitalization and close monitoring. A technological upgrade of monitoring methods in hospital wards may be part of the solution. In all these respects, telemedicine has proven to be a valuable ally during this pandemic. A unified European and world policy has been lacking in this regard, leaving the solution to the discretion of each country, which is influenced by the sociocultural context and the possible repercussions on public opinion.

There is a need for safety protocols, maintenance personnel, and experts dedicated to the training of health workers. Managing and organizing computerized health data requires specific professional skills: this is the field of the Data Scientist, who should be involved since inception in the data mining life cycle: collection, preparation, exploration, analysis, prediction, prescription, and reporting [32].

Clinicians of all specialties, in each country, have faced an unknown disease, adopting therapeutic protocols that they deemed to be suitable to fight the new disease and resorting to drugs and medical devices that were already in their possession. In this climate of uncertainty, the exchange of new diagnostic, instrumental, and therapeutic knowledge between health workers from various countries is of fundamental importance.

AI and new medical technologies are powerful tools that should be closely supervised to make sure that their application to the healthcare field is in accordance with current regulations. These devices are leading the field toward Medicine 2.0. All these instruments are going to take a part in daily clinical practice, and they have the potential to connect the world, making healthcare more inclusive, diffuse, and at the same time tailored to the patients.

New technologies have an essential role in the humanization of hospital care, which consists in not only providing patients with excellent medical care through the appropriate treatment and supportive measures but also discussing the quality, quantity, and type of health care with patients and their family, and in providing emotional and psychological support for patients, families, and staff [59].

It is now known that hospitalized COVID-19-positive patients must remain isolated from the outside world. Patients can interact only with healthcare professionals. In this era, the interaction with healthcare personnel has become more complicated because of the physical screens placed between healthcare workers and patients, such as masks, sanitary suits, and personal protection devices. Patients have a restricted way of communicating with their relatives and medical staff. Furthermore, all the medical protective devices make healthcare workers indistinguishable. Patients cannot understand whether the people they are talking to are nurses, doctors, or other healthcare personnel, making them feel disoriented and anxious. Despite this, it was possible to adopt some modern and technological solutions to give comfort to patients. A small laptop or smartphone allows communications with the healthcare workers, listening to music therapy sessions, or video-calling or recording a diary (intensive care diaries), or contact between patients and their families outside the intensive care units. During the whole period of hospitalization in intensive care or in COVID-19 wards, relatives received information about their sick family members from the doctors via a phone call, approximately once or twice a day. When patients were intubated, family members could no longer see them until they were discharged. Unfortunately, the high case fatality rate of Coronavirus implied that some families could not see their relatives anymore, after they were admitted to the hospital. The use of electronic devices, such as tablets, and the possibility to make video calls would allow families to talk to and see their sick relatives. Obviously, these tools should also be easy to use for non-IT professionals.

## Data Availability

Data sharing is not applicable to this article as no new data were created or analyzed in this study.
